# Exploring the therapeutic and anti-tumor properties of morusin: a review of recent advances

**DOI:** 10.3389/fmolb.2023.1168298

**Published:** 2023-05-09

**Authors:** Amna Hafeez, Zeeshan Khan, Muhammad Armaghan, Khushbukhat Khan, Eda Sönmez Gürer, Ahmad Faizal Abdull Razis, Babagana Modu, Zainab M. Almarhoon, William N. Setzer, Javad Sharifi-Rad

**Affiliations:** ^1^ Atta-Ur-Rahman School of Applied Biosciences, National University of Sciences and Technology, Islamabad, Pakistan; ^2^ Department of Pharmacognosy, Faculty of Pharmacy, Sivas Cumhuriyet University, Sivas, Türkiye; ^3^ Department of Food Science, Faculty of Food Science and Technology, Universiti Putra Malaysia, Serdang, Selangor, Malaysia; ^4^ Natural Medicines and Products Research Laboratory, Institute of Bioscience, Universiti Putra Malaysia, Serdang, Selangor, Malaysia; ^5^ Department of Biochemistry, Faculty of Science, University of Maiduguri, Maiduguri, Borno State, Nigeria; ^6^ Department of Chemistry, College of Science, King Saud University, Riyadh, Saudi Arabia; ^7^ Aromatic Plant Research Center, Lehi, UT, United States; ^8^ Department of Chemistry, University of Alabama in Huntsville, Huntsville, AL, United States; ^9^ Facultad de Medicina, Universidad Del Azuay, Cuenca, Ecuador

**Keywords:** morusin, natural compound, anti-cancer activity, polyphenol, resistant malignancies

## Abstract

Morusin is a natural product that has been isolated from the bark of *Morus alba*, a species of mulberry tree. It belongs to the flavonoid family of chemicals, which is abundantly present in the plant world and is recognized for its wide range of biological activities. Morusin has a number of biological characteristics, including anti-inflammatory, anti-microbial, neuro-protective, and antioxidant capabilities. Morusin has exhibited anti-tumor properties in many different forms of cancer, including breast, prostate, gastric, hepatocarcinoma, glioblastoma, and pancreatic cancer. Potential of morusin as an alternative treatment method for resistant malignancies needs to be explored in animal models in order to move toward clinical trials. In the recent years several novel findings regarding the therapeutic potential of morusin have been made. This aim of this review is to provide an overview of the present understanding of morusin’s beneficial effects on human health as well as provide a comprehensive and up-to-date discussion of morusin’s anti-cancer properties with a special focus on *in vitro* and *in vivo* studies. This review will aid future research on the creation of polyphenolic medicines in the prenylflavone family, for the management and treatment of cancers.

## Introduction

No nation is immune to the devastating effects of cancer. It is one of the biggest killer worldwide (Prager et al., 2018). Most of the time, cytotoxic drugs are used to stop the growth of malignant cancer cells. These drugs stop cancer cells from dividing, but they also cause severe side effects that are not intended. Because of this, a significant amount of research is currently being conducted on the use of chemicals obtained from natural origins as powerful inhibitors of cancer. These compounds have their origins in a variety of conventional therapeutic practices.

A prenylated flavonoid named morusin is one example of such a compound found in nature. Morusin has been studied for its purported ability to fight infections, oxidative stress, and inflammation (Davatgaran-Taghipour et al., 2017). Numerous studies have demonstrated how effective morusin is as a potent anti-cancer drug. Morusin has a wide range of pharmacological effects, including defense against neuroblastoma cell death caused by nitric oxide, the capacity to scavenge superoxide anion radicals, anti-diabetic activity, adipocyte differentiation, and more. Morusin’s cytotoxic effects have been mainly seen in various cancer cell lines, such as those of breast adenocarcinoma, colorectal adenocarcinoma, gastric cancer, etc. Morusin’s ability to suppress Nuclear factor kappa B (NF-κB) and Signal transducers and activators of transcription 3 (STAT-3) activation in prostate, pancreatic, and liver cancer cells has been described in recent research (Agarwal et al., 2019). In the last decade, the number of *in vitro* and *in vivo* studies, focusing on anti-tumor properties of morusin, have increased and morusin is quickly establishing its role as a promising anti-cancerous drug target. This is the reason, morusin has been made the focus of this review.

The study’s overarching purpose is to get a deeper understanding of one of the most effective compounds that has shown promising data regarding inhibition of several forms of cancer. In addition, this study will aid in consolidating the dispersed data regarding the efficacy of morusin as a therapeutic agent into a unified database, where knowledge gaps may be identified and plans to be made in order to fill them. All the *in vitro* and *in vivo* studies performed regarding the anti-tumor properties of morusin have been included within this review. Furthermore, this scientific review intends to organize the existing state of understanding of the positive features of morusin to bring it to the awareness of investigators and healthcare doctors for future application in the management and treatment of certain ailments. The findings of this review are of critical importance as morbidity and mortality rates of cancers are increasing exponentially and there is a dire need to identify novel therapeutic targets.

### Review methodology

**FIGURE 1:**
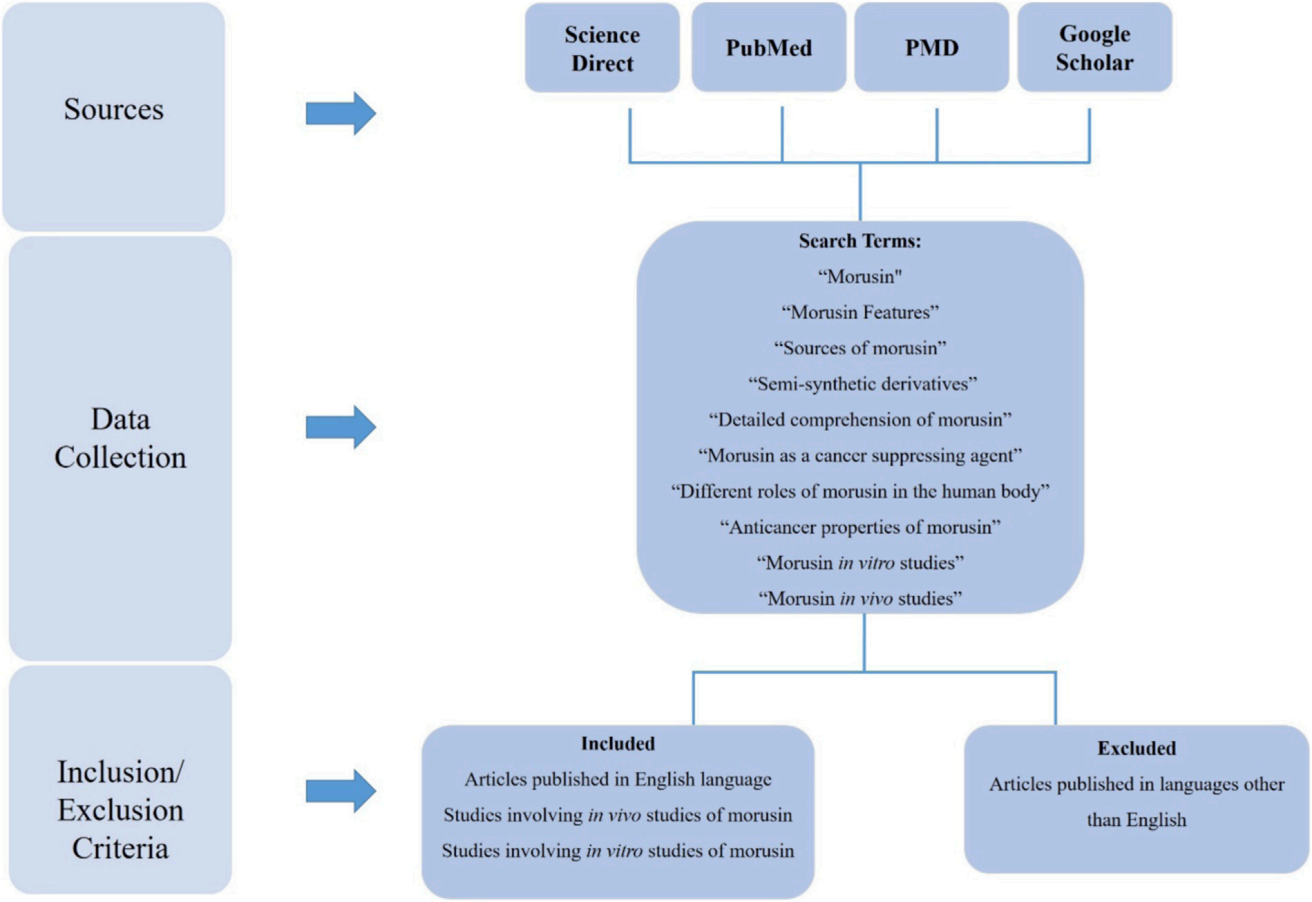
Schematic diagram of review methodology.

Science direct, PubMed, the European Preliminary Material Database (PMD), and Google scholar were used to locate the relevant publications. Key terms such as “Morusin features” “semi-synthetic derivatives,” “detailed comprehension of morusin” “sources of morusin,” “morusin as a cancer suppressing agent,” “Morusin” “different roles in the human body”, “Anti-cancer properties of Morusin”, “Morusin *in vitro* studies”, “Morusin *in vivo* studies” etc. were used to compile the data. In addition, when we were analyzing the data, we omitted any publications that did not support the English language and were not published in credible journals. Schematics of the study are shown in Figure 1.

### Plant sources of morusin

Numerous plant species that belongs to family Moraceae (sometimes called the fig family or the mulberry family) are good sources of morusin. This family has more than 1,100 species over around 38 genera. The majority of their distribution is centered in the tropics and subtropics, while the temperate zones only host a smaller percentage (Nair et al., 2021). After reviewing the available literature, a number of plant species were found to be credible sources of morusin (Table 1).

**TABLE 1:** Plant sources of Morusin.

Plant name	Common name	Plant height	Cultivation area/Naturalized area	Presence of morusin	Plant picture	References
*Morus alba* L.	Common mulberry, silkworm mulberry, white mulberry, and silkworm mulberry	10–20 m (33–66 feet)	India, Türkiye, United States, Argentina, Kyrgyzstan, Australia, Mexico, Iran, and many more	Root Bark	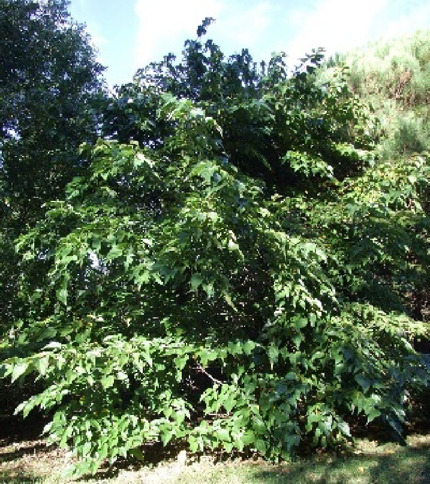	(Li et al., 2018; Patel et al., 2021)
*Morus nigra* L	Black mulberry or Blackberry	9–12 m (30–40 feet)	Southern Asia and the Iberian Peninsula	Root Bark	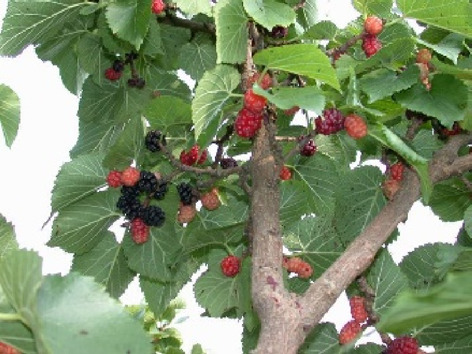	(Zoofishan et al., 2018)
*Morus notabilis* C.K. Schneid	Mulberry tree	Between 1,300 and 2,800 m (4,300 and 9,200 ft)	Chinese provinces of Yunnan and Sichuan	Plant’s twigs	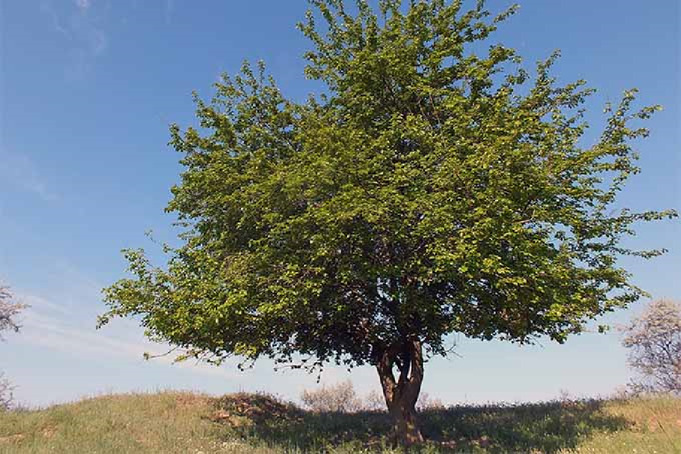	(Yan et al., 2020)
*Morus lhou* Koidz	_	8–15 m	East Asia	Root bark	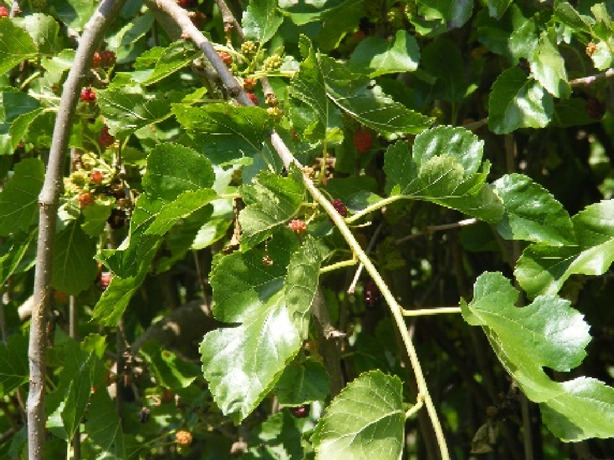	(Jeong et al., 2009; Kim et al., 2011)
*Morus australis* Poir	Korean mulberry and Chinese mulberry	7.5 m	East and Southeast Asia	Cortex	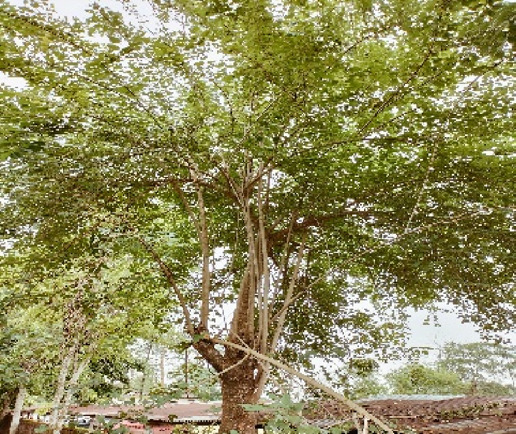	Syah et al. (2008)
*Artocarpus tonkinensis* A. Chev	_	16 m	Southeast Asian and Pacific origin	Roots	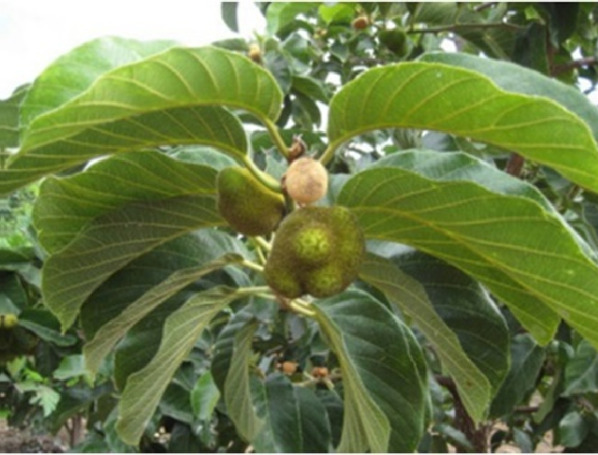	(Jagtap and Bapat, 2010; Ma et al., 2010)
*Artocarpus altilis* (Parkinson) Fosberg	Breadfruit	5–8 m	Maluku Islands, New Guinea, and the Philippines	Stem bark	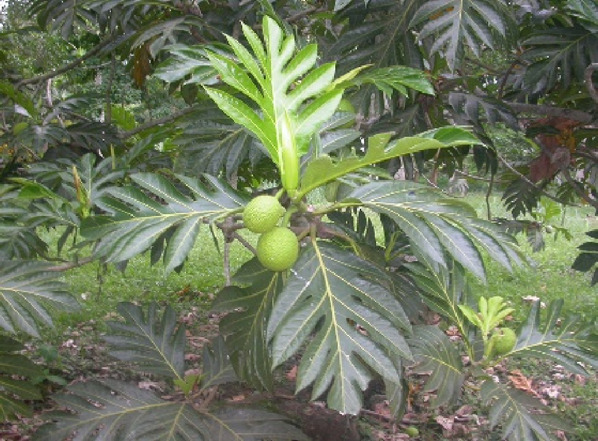	(Shamaun et al., 2010; Zhang and Shi, 2010; Wan et al., 2014)

### General characterization of morusin

Natural prenylflavonoids are a promising class of compounds because they exhibit a diverse range of pharmacological activity. The flavonoids include morusin, which is found in berries (Chen et al., 2018). The location and the quantity of functional groups connected to the flavone backbone affect the compound’s bioactivities. While prenylation makes compounds more lipophilic, increasing their affinity for cell membranes, it also reduces their bioavailability and their ability to enter the bloodstream (Hakim et al., 2006).

**FIGURE 2:**
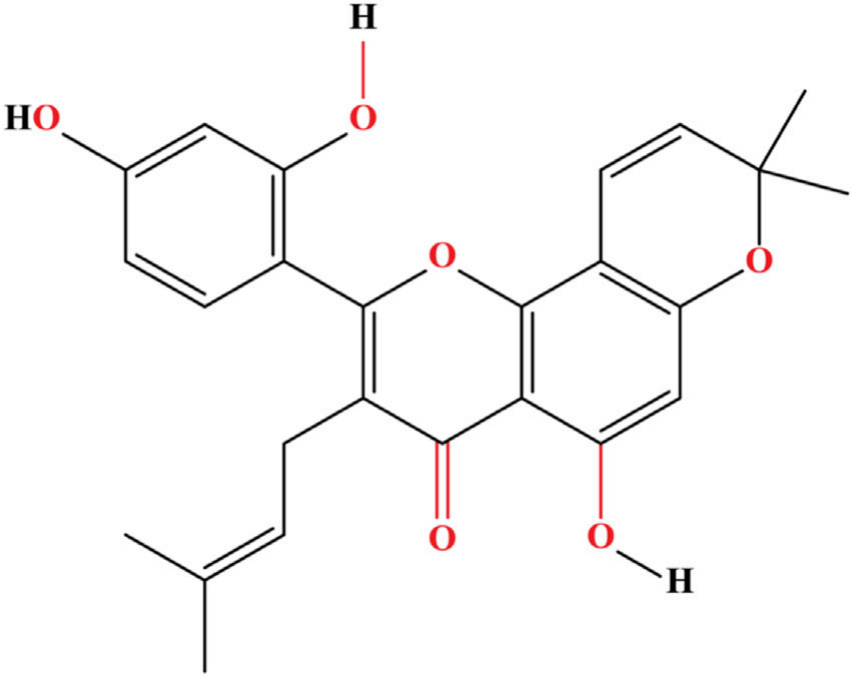
The flavonoid morusin contains a flavone core substituted at 5, 2′, and 4′by hydroxy groups, a prenyl core at 3, and a 2,2-dimethyl pyran core at 7 and 8.

Prenylations at the C-3 and C-7 positions—possible that certain places in the flavone chain of morusin are responsible for the significant cytotoxicity of the molecule when it comes to murine P-388 cells. Kuwanon C, in contrast to morusin, possesses a free prenyl group at the C-8 position. The structure of morusin is given in Figure 2. This greatly enhances the antagonistic effect against *ß*-secretase as well as the anti-bacterial activity against *Escherichia coli* and *Salmonella typhimurium*, while marginally reducing the cytotoxic effects. Morusin does not possess this feature (Cho et al., 2011). The reduction in inhibitory activity of morusin against tyrosinase and α-glucosidase was attributed to the cyclization of the prenyl group at position 7 of morusin (Yan et al., 2020). Morusin has the potential to allosterically regulate the activity of a number of enzymes, including cyclooxygenase-2, acetylcholinesterase (AChE), lipoxygenases, pancreatic lipase, epidermal growth factor receptor, cytochrome P450, matrix metalloproteinases (MMP-9 and MMP-2), and uridine 5′-diphospho glucuronosyltransferase (UDP-glucuronosyltransferase) (Shi et al., 2016a).

Uncoupling its antioxidant and anti-inflammatory properties reflects the fact that each carbon residue appears to have a unique role in regulating biological processes. Noncompetitive inhibitory features toward AChE were seen, for instance, when morusin was prenylated at C-3; in contrast, the inhibition kinetics of non-prenylated flavonoids were variable (Kim et al., 2011).

Morusin’s selective inhibition of particular oxygenases was also dependent on whether or not it had been hydroxylated at its C-5′ position. Because it lacks artonin E’s 5′-hydroxyl group, morusin is a weaker 5-lipoxygenase inhibitor. When tested against a panel of lipoxygenases, morusin showed potent inhibitory activities; however, artonin E, which is formed by hydroxylation of morusin at its C-5′ position, showed that 5-lipoxygenase inhibition that was slightly higher. Morusin forms hydrogen bonds with Cytochrome P450 3A4 (CYP3A4) at its C-5 and C-2′ pyran ring oxygens, and its B ring structure interacts with Phe108 of CYP3A4 via a π-π interaction, as shown by a molecular docking study (Shi et al., 2016b).

Docking studies have shown that the 2′- and 4′-hydroxy groups of the B ring structure of morusin establish hydrogen bonds with Val127 of the B and F chains in arachidonate 5-lipoxygenase (5-LOX). The hydrophobic curve created by Val127, Ala128, Leu124, Leu135, and Phe131 in the 5-LOX active site coincides with the location of the 3-prenyl group in morusin, demonstrating the importance of morusin’s hydroxylation and prenylation in recognizing the protein target (Agarwal et al., 2019). Screening for gamma-aminobutyric acid (GABA) transporter 1 ligands was performed using protein-based molecular modelling and docking analysis. Hydrogen bonding between morusin and GABA transporter 1’s Ser396 and Tyr140 and implied that morusin is the most promising potential ligand for this protein (Bankupalli et al., 2020).

### Bioavailability of morusin

Bioavailability refers to the rate and extent of absorption of the active moiety (drug or metabolite) into systemic circulation and ultimately the site of action. The bioavailability of a medicine is highly dependent on the properties of the dosage form, which are in turn impacted by the design and production of the dosage form (Fernandes and Jozala, 2022).

However, the conventional Chinese drug frameworks pharmacy directory and assessment framework has set a target value of over 20%, morusin has an oral bioavailability of 11.52% (Zhou et al., 2021). As a hydrophobic hydroxyflavone, morusin is almost insoluble in water and therefore has a very low solubility in body fluids (Agarwal et al., 2019).

### Semi-synthetic derivatives

Morusin, also known by its IUPAC designation 2-(2,4-dihydroxyphenyl)-5-hydroxy-8,8-dimethyl-3-ol, has the molecular formula C_25_H_24_O_6_ and the molecular weight 420.5 g/mol (3-methylbut-2-en-1-yl)-4H, 8H-pyrano [2,3-f] chromen-4-one. It is chemically classified as a flavone and has characteristic hydroxyl groups on carbons 5, 2′, and 4′. Morusin structural analogues can be obtained by replacing the unattached hydroxyl groups with other functional groups (Davatgaran-Taghipour et al., 2017).

### Current medical applications

#### Antioxidant activity

Antioxidants are found naturally in polyphenols. Because they include free hydroxyl groups, they also have antioxidant capabilities. Free radicals can cause damage to an organism, but antioxidants help prevent that. Polyphenols’ activity stems from their ability to scavenge reactive oxygen species, chelate transition metals in order to prevent the formation of hydroxyl radicals, protect antioxidants from oxidation, and inhibit lipoprotein oxidation (Treml et al., 2021).

Mulberry leaves contain antioxidants that may reduce the oxidative stress and cell damage caused by free radicals. Abbas et al. have shown, by the 2,2′-azino-bis(3-ethylbenzothiazoline-6-sulfonic acid) (ATBS) assay, that prenylation and isoprenyl cyclization reduce the antioxidant activity of chemicals identified in *Morus nigra*. The capacity of morusin to scavenge free radicals was found to be 61.34% lower than that of kuwanon C, which possesses two free isoprenyl groups at C-5 and C-8. This is because the hydroxyl group at C-7 in morusin is cyclized to the pyran ring (Martins et al., 2021).

#### Efficacy against bacteria and protozoa

The prenylated flavones, flavanones, and chalcones found in nature have been shown to have antibacterial effects. Similarly, studies have demonstrated that morusin is efficient against both bacteria and fungi. Morusin’s antibacterial effects were originally reported in 2019 by Wu et al. (2019), who showed that it was especially effective *versus* Gram-positive bacteria of the *Staphylococcus*, *Bacillus*, and *Enterococcus* genera. On the other hand, Gram-negative bacteria have consistently shown no sensitivity to morusin. This may result from the fundamental dissimilarity between Gram-positive and Gram-negative cell membranes.

Using *S. epidermis* ATCC 12228 and *S. aureus* strains, researchers validated morusin’s antibacterial activity against Gram-positive bacteria. A team of scientists discovered by transmission electron microscopy (TEM) and scanning electron microscopy (SEM) that morusin damages the cell wall and membrane of bacteria by altering the expression of genes associated in the cell’s phosphatidic acid production pathway (Zhou et al., 2021). A type of fats known as phospholipids stabilize cell membranes, with phosphatidic acid serving as the major substrate in biogenesis. The fatty acid profile of *S. aureus* cells in the presence of morusin also changed, which may be related to the inhibition of the acyltransferases action that catalyze the lipid transformation within cells. Morusin had a minimum inhibitory concentration (MIC) of 14.9 mol/L against both *S. aureus* ATCC 25923 and *S. aureus* ATCC 6538. Although the MIC values of morusin were higher as compared to ampicillin but the inhibitory effect of morusin was higher (Pang et al., 2019). As a result, it is possible that plasma membrane disruption caused by morusin, has an indirect effect on cell viability in bacteria. These findings are in agreement with the notion that prenylations i. e., addition of a prenylated side chain (i.e., prenyl, geranyl and lavandulyl) attached to the flavonoid skeleton can boost morusin’s lipophilicity and, consequently, their affinity for bacterial cell membranes (Sohn et al., 2004; Aelenei et al., 2020). Anti-malarial properties of morusin again the protozoan *Plasmodium falciparum* have also been observed. Researchers observed that morusin isolated from *Artocarpus altilis* inhibited the growth of human malarial parasite at IC_50_ value of 4.5 µM (Boonphong et al., 2007). These findings can help in the development of ant-protozoan therapies in the future.

#### The anti-inflammatory properties

Morusin possesses anti-inflammatory attributes, the genomic processes of which involve the inhibition of cyclooxygenase (COX) and lipoxygenase (LOX) activity. Cyclooxygenase is an enzyme that plays a crucial part in the biosynthesis of prostanoids, such as prostaglandins, which are produced by the modification of arachidonic acid. Inflammatory conditions, malignancies, and degenerative disorders are all associated with an increase in the production of this enzyme. One of the most notable aspects of morusin would be its effect on nuclear regulator NF-κB and inducible nitric oxide synthase (iNOS), in addition to its ability to inhibit cyclooxygenase and lipoxygenase activity (Jia et al., 2020).

Morusin has been shown to reduce pure COX-1, 5- and 12-LOX from swine leukocytes at concentrations between 0.1 and 10 mM. Chi’s research demonstrated that morusin inhibited COXs and LOXs with IC_50_ values more than 100 μM. Morusin substantially suppressed iNOS enzyme activity at doses larger than 10 μM in lipopolysaccharide-activated murine macrophage RAW 264.7 lines, as discovered by Cheon et al. (2000). Similar results were reported by Tseng et al. (2018), who said morusin offered liver protection.

#### The ability to prevent convulsions

There are a number of potential clinical origins for epilepsy, which are a type of neurological illness characterized by transient disruptions in brain activity and connected with aberrant electrical discharges in neurons (Royer et al., 2022). Based on epidemiological studies, epilepsy has been shown to be the second most common kind of neurological disorder. Seizures in epilepsy are thought to originate from an imbalance between exuberant and regulatory signals in the nervous system, from a neurochemical standpoint. This discord may be caused by a number of factors, including shifts in glutamatergic and GABAergic neurotransmission, faulty ion channels and pumps, and an unbalanced state of cellular metabolism and energy production (Beghi, 2020).

Prenylated neuroflavanones such as prenylnaringenin are effective for modulating the GABA_A_ receptor. Morusin’s capacity to prevent isoniazid- and maximum electroshock-induced convulsions in live mice was investigated at two dosages, 5 and 10 mg/kg. Morusin, at a dosage of 20 mg/kg (ED_50_), prolonged the maximum electroshock seizure assessment and delayed the onset of convulsions and tonic hind limb extension. The death rate of mice treated with morusin, experiencing isoniazid- and electroshock-induced convulsions was also drastically decreased. Also, morusin (5 and 10 mg/kg) raised brain GABA levels in the tested rats (Nadhiya, 2019; Chen et al., 2021a).

#### Clinical use of morusin for memory impairments

Traditional Chinese medicine makes use of the white mulberry (Morus alba L.). Compounds contained in it have been shown to safeguard neurons. According to a study conducted in rats performed by Gupta et al. (2017) in 2017, morusin can prevent the memory impairment caused by aluminium trichloride. Memory impairment in rats was improved by lowering the acetylcholinesterase activity induced by AlCl_3_ and oxidative stress levels within brain.

Kim *et al.* found that morusin and its C-3 isoprenyl derivatives, including cyclomorusin, neocyclomorusin, and kuwanon C, inhibited acetylcholinesterase, the enzyme responsible for breaking down acetylcholine into choline and acetic acid, in a non-competitive manner. Both acetylcholinesterase and butyrylocholinesterase were inhibited at concentrations of 36.4% and 24.8%, respectively, by morusin. The effectiveness of inhibiting substances like those listed above increased with concentration. Morusin and its derivatives may be useful agents in the battle against neurodegenerative illnesses like Alzheimer’s disease because of acetylcholinesterase (EC 3.1.1.7) action, which hydrolyzes the ester bonds in neurotransmitters like acetylcholine to stimulate cholinergic neurotransmission (Panek-Krzyśko and Stompor-Gorący, 2021).

#### Action against hyperglycemia

The modern world still struggles with obesity and its associated health issues. Prenylated polyphenols found in plants have been shown to have anti-diabetic action in several studies. Both advanced glycation end-product (AGE) inhibitors and non-competitive aldose reductase 1B1 inhibitors have been discovered (AKR1B1 and AKR1B10). Standardized plant extracts were tested for their *in-vitro* biological activity in the management of type 2 diabetes. One such extract included morusin and its derivatives from the *Morus alba* plant. Researchers looked at how well the extract blocked the development of AGEs and aldose reductase (Fakhree and Nazemiyeh, 2012).

Extracts of several sections of the mulberry, *Morus alba*, have been shown to contain prenylated flavonoids with α-glucosidase inhibitory action (Chen et al., 2018). The root bark (*Cortex Mori*) extract was the most potent because it contained the largest quantity of prenylated flavonoids as measured by HPLC.

#### Influence of morusin on lipid metabolism

When morusin was employed, mouse cell line showed a 51% drop in triglyceride (TG) deposition and a 70% drop in cytoplasmic glycerol-3-phosphate dehydrogenase (GPDH) action (Yang et al., 2011). Furthermore, morusin inhibited nitric oxide production by LPS-stimulated RAW 264.7 cells with an IC_50_ of 10.6 μM (Yang et al., 2011). This property of morusin could play a potential role in development of anti-obesity therapies. In another study it was observed that morusin promotes lipid accumulation in cancer stem cells and predisposes them to differentiate into adipocyte-like cells. The adipogenic proteins PPAR, adipsin, aP2 and perilipin were upregulated as well as the content of lipids in glioma stem cells (GSC) after morusin therapy (Guo et al., 2016). This ability of morusin could serve as a potential therapy for cancers by trans-differentiating cancer stem cells into adipocytes.

#### Action against spasms

Muscle disorder diseases of the digestive system and asthma both cause constriction, respectively, in the gastrointestinal and respiratory systems. As resistance to standard treatments becomes more common, researchers are looking for alternative treatment methods involving the use of naturally occurring chemicals that could have antispasmodic effects. Antispasmodic components of *Morus nigra* were studied by Zoofishan et al. (2018) in 2018. Weak relaxant action was shown for morusin and its equivalents in rat ileum smooth muscles, while moderate activity was observed for rat tracheal smooth muscles.

#### Efficacy against viruses

In a study performed in 2020, Thabti et al. (2020) looked into the antiviral properties of aqueous extracts of *Morus* spp. Since coronaviruses are a major cause of respiratory tract infections. It has been shown that natural chemicals, such as kuwanon C, found in the leaves and stem bark of *Morus alba*, have antiviral activity against human coronavirus HCo-V-229E. It was also shown that most of the extracts tested strongly reduced the replication of human poliovirus, parechovirus, and echovirus (Chen et al., 2018).

#### Actions against osteoporosis

Bone formation and remodeling during childhood and adolescence, bone remodeling in adult organisms, fracture healing, and the maintenance of calcium and phosphate balance are all important for whole-body health. In osteoporosis, bone cells (osteoclasts, osteoblasts, and others) fail to respond to signals from one another, leading to a breakdown in the normally tight connection between bone resorption and bone creation (Song et al., 2022). Bone resorption, turnover, and the frequency of reshaping locales all rise with age, whereas bone formation processes fall off. Consequently, efforts are being made to discover novel methods for halting bone density decline and stopping the onset of osteoporosis. Isoprenyl-containing natural compounds have emerged as a potential strategy for treating osteoporosis (Lim et al., 2018; Chen et al., 2021b).

#### Efficacy as an anti-nociceptive

The anti-nociceptive system in mice, which is in charge of regulating pain transmission, was studied *in vivo* by De Souza et al. (2000) in 2000. They extracted morusin from the root bark of *Morus nigra*. Those findings demonstrated that morusin shows promise as a pain-modulating agent. Compared to commonly used analgesics like aspirin and paracetamol, morusin was shown to be more efficient in modulating pain perception. The anti-nociceptive effects of morusin could be counteracted by naloxone, an opioid receptor antagonist and one of the painkillers.

#### Inhibition of enzyme activity

Chaita et al. (2017) looked into the mechanism through melanins (skin pigments) are made in melanocytes. *Morus alba* and a few other plant extracts were used to obtain the active components. Melanocytes use tyrosinase to synthesize melanin, which is then packaged into melanosomes. Tyrosinase is a crucial enzyme in the biosynthesis of melanin, since it catalyzes the transition from tyrosine to L-3,4-dihydroxyphenylalanine (L-DOPA) and from L-DOPA to dopaquinone.

Skin pigmentation serves to shield the body from harmful ultraviolet radiation from the Sun, which has been linked to skin cancer and the ageing process. The mouse melanoma cell line B16F10 was employed in the study by Chaita et al. (2017) *.* The methanolic extract of *Morus alba* was the most potent inhibitor of intracellular tyrosinase (IC_50_ = 0.4 g/mL) among the investigated plant extracts. Enzyme activity was decreased by 49.9 percent when morusin was added at a concentration of 300 μM. *Morus alba’s* 2,4,3′-trihydroxydihydrostilbene (IC_50_ = 0.8 μM) was the most potent tyrosinase inhibitor identified.

#### Anti-nephritis activity of morusin

The inflammation of the glomeruli is known as glomerulonephritis, and it is a frequent condition of the urinary system. Morusin and its derivatives, including glabridin, were studied by Fukai et al. (2003) in 2003, for their potential anti-nephritis effects. Biochemical indicators such urine protein excretion, total cholesterol, serum creatinine, and blood urea nitrogen were studied in a mouse model of glomerular disease. Scavenging activity for superoxide anion radicals (O_2_·^–^) and autoxidation (radical intensity under alkaline conditions) efficiency were measured using Electron Spin Resonance (ESR) spectroscopy. Radical intensity is the concentration of free radicals or reactive oxygen species present at a given time that react with proteins and result in cellular and tissue damage. The ability of morusin to reduce the concentration of free radicals was measured using ESR spectroscopy. Oral dosing at 30 mg/kg per day was used for the substances under investigation. The ESR spectra demonstrated that morusin improved the radical intensity of sodium ascorbate and had a minor scavenging action against the superoxide anion radical. Serum creatinine and blood urea nitrogen levels were also reduced, along with urinary protein excretion.

#### Anticancer properties of morusin

**FIGURE 3:**
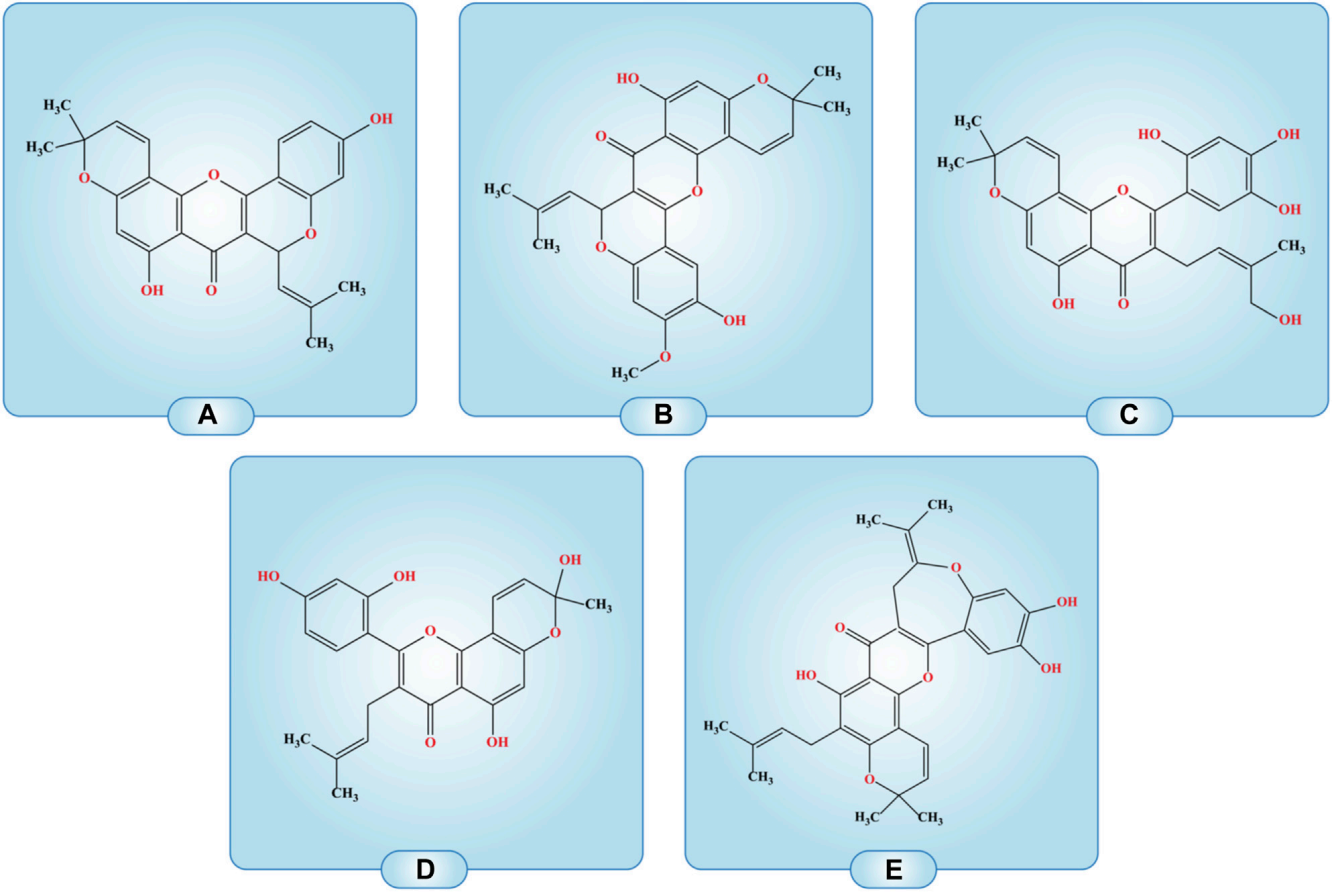
Compounds with the structural arrangement similar to morusin are; **(A)** cyclomorusin A, **(B)** Cycloartomunin, **(C)** 5-hydroxy-3-(4-hydroxy-3-methyl-but-2-enyl)-8,8-dimethyl-2-(2,4,5-trihydroxy-phenyl)-8H-pyrano [2,3-f]chromen-4-one, **(D)** 2-(2,4-Dihydroxyphenyl)-5-hydroxy-8-(hydroxymethyl)-8-methyl-3-(3-methylbut-2-en-1-yl)-4H, 8H-pyrano [2,3-F]chromen-4-one, **(E)** Isocycloheterophyllin.

Cancer is the leading cause of morbidity and mortality worldwide and millions of people are affected by the many subtypes of cancers. Cancers with the highest cases worldwide include breast cancer, (2.26 million cases, lung (2.21 million cases), colon and rectum (1.93 million cases), prostate (1.41 million cases), skin (non-melanoma) (1.20 million cases) and stomach (1.09 million cases) (Siegel et al., 2023; Harinee et al., 2022). There is a need to find novel treatment options for cancer patients. Plant sources have proven to be promising research avenue for scientists. The anticancer effects of morusin have been demonstrated. Breast, ovarian, and colon cancer development have all been shown to be slowed by morusin in laboratory settings. There is evidence that morusin can cause cancer cells to commit suicide by way of a process called apoptosis. Although morusin has anticancer properties, the exact processes by which it works are not yet understood, but it is thought to involve the inhibition of significant signaling pathways for tumor growth and survival (Table 2; Figure 3). Further research is needed to fully understand the potential of morusin as a cancer therapeutic agent and to determine the optimal dosage and administration for its use in cancer treatment.

**TABLE 2:** Morusin activity in different cancers.

Cancer	Targeted pathway	Impact of morusin	References
Hepatocellular carcinoma	STAT3 Pathway	G1 arrest	Cho et al. (2021)
Renal cell carcinoma	MAPK Pathway	Lowering ERK levels	Yang et al. (2020)
Colorectal cancer	PI3K/Akt Pathway	Inactivation of Akt	Zhou et al. (2021)
Nasopharyngeal carcinoma	ERK1/2 Pathway	Suppressing the expression of MMP-2	Huang et al. (2020)
Breast cancer	STAT3 Pathway	Suppressing Survivin and inducing Bax proteins	Kang et al. (2017)
Prostate cancer	STAT3 Pathway	Inhibition of phosphorylation of STAT3	Lim et al. (2015)
Gastric cancer	MAPK Pathway	Down-regulating c-Myc and CDKs	Wang et al. (2017)
Glioblastoma multiforme	STAT3 Pathway	Downregulation of Bcl-2 and upregulation of Bax	Guo et al. (2016)

**Abbreviations:** STAT3, Signal transducers and activators of transcription 3, MAPK, Mitogen-activated protein kinase, Bcl-2, B-cell lymphoma 2, CDK, Cyclin-dependent kinases, C-Myc, Cellular myelocytomatosis, BAX, Bcl-2-associated X protein, MMP, matrix metalloproteinase.

##### Hepatocellular carcinoma

One of the most commonly occurring diseases in the world, liver cancer is on the rise in Western nations. It affects men 2.3 times more commonly than women globally, with a death to incidence ratio of 0.91, and accounts for 72% of newly diagnosed cases in Asia (Ferlay et al., 2019). To a greater extent than any other primary liver cancer, hepatocellular carcinoma (HCC) is linked to long-term liver damage from hepatitis B or C virus infection, alcoholism, or the metabolic syndrome (Sangro et al., 2021). Synchronic carcinogenesis or early spread inside the liver, HCC is frequently multinodular at diagnosis and has a special propensity to grow inside blood vessels, penetrating the portal or hepatic veins. Synchronic carcinogenesis is the phenomenon of development of multiple tumors simultaneously which is a common factor in HCC (Gisder et al., 2022). Although chemotherapy, radiation, immunotherapy, and surgery have long been used to treat HCCs, antiglycolytic drugs like 3-bromopyruvate may also be utilized alone or in combination with other traditional anticancer drugs like sorafenib (Wu et al., 2021a). Additionally, AMPK, PI3K/Akt pathway, HIF-1, and c-Myc have been identified as proteins associated with aerobic glycolysis in hepatocellular cancer. In a study, glycolysis and G1 arrest were linked to the anticancer mechanism of morusin in Huh7 and Hep3B cells. Morusin strongly demonstrated cytotoxicity in Huh7 and Hep3B cells using the MTT and CCK-8 assays, and it reduced the number of colonies in these cells, indicating that it has anticancer potential. Likewise, by blocking the IL6/STAT3 signaling pathway, morusin caused apoptosis and prevented angiogenesis in hepatocellular cancer cells (Cho et al., 2021).

##### Malignant tumor of the kidney (renal cell carcinoma)

Pernicious tumors like renal cell carcinoma (RCC) are on the rise, and they’re responsible for 2% of all cancer cases and deaths globally (Padala et al., 2020). In the industrialized world as a whole, the incidence of kidney and renal pelvis malignancies has sharply grown over the past few decades. In the United States, it has increased by more than 100% since 1975. The cortex of the kidney is where most RCCs develop; it contains the glomerulus, tubular apparatus, and collecting duct. Renal pelvic cancers have a similar histology and clinical behavior to bladder cancer (Padala et al., 2020). In a research, the cytotoxic and anti-tumor properties of morusin were examined in three renal cell carcinoma (RCC) cells 769-P, 786-O, and OSRC-2 (Yang et al., 2020). Inhibition of RCC cell proliferation and migration was dose and time-dependently induced by morusin administration. Subsequent experiments demonstrated that morusin may induce apoptotic and cell cycle arrest in the G1 phase by elevating the expression of Bax and cleaved-caspase 3 and inhibiting the expression of Bcl-2, CDK4, CDK6, and Cyclin D1 (Yang et al., 2020). These data showed that morusin might be a valuable therapeutic option for RCC. The cytotoxic effects of morusin were mediated through its interference with MAPK signaling. It raised P-38 and P-JNK and lowered P-ERK in 769-P, 786-O, and OSRC-2 cell lines. Morusin was found to inhibit the growth of RCC tumorus in xenograft experiments using nude mice (Yang et al., 2020).

##### Colorectal cancer

Colorectal cancer is the third most prevalent malignant tumor and the second most lethal type of cancer (CRC). CRC accounted for around 10% of all new cancer diagnoses and deaths in 2018, with an estimated 1.8 million new cases being diagnosed (Bray et al., 2018). Almost 2.5 million more cases of CRC are expected to be diagnosed by 2035 (Schatoff et al., 2017). Advances in both primary and supplementary treatments have contributed to a rise in CRC patients’ median survival times. Surgical removal of the primary tumor and any distant metastases is the gold standard for treating CRC. Radiation treatment and chemotherapy are the mainstays of care for malignancy in patients with inoperable or surgically-refractory tumorus (Wolf et al., 2018). Maximum tumor shrinkage and prevention of cancer’s spread and development are priorities for these individual (Xie et al., 2020).

Morusin was found to prevent the beginning of colorectal cancer spheroid formation as well as the proliferation of colorectal HCT116 sphere cells. Morusin also reduced Oct4 and Nanog expression, two stemness markers (Zhou et al., 2021). The study illustrated the potential molecular pathways of morusin’s actions on colon cancer stem cells (CCSCs). First, GSK-3 (Chen et al., 2000), another signaling target of the canonical Wnt/-catenin pathway, is downregulated by activated Akt. It is understood that the Wnt/-catenin pathway has activating mutations in almost every case of colon cancer and is crucial for CCSCs (Prasetyanti et al., 2013). The tumor-initiating potential is activated and CSC development is maintained by the constitutive activation of the Wnt/-catenin pathway. Significantly more GSK was expressed and less *ß*-catenin was active due to the inactivation of Akt caused by morusin. Morusin was discovered to stop the onset of colorectal cancer spheroid formation as well as the proliferation of colorectal sphere cells (Zhou et al., 2021). It also decreased the expression of the downstream proteins, c-Myc, survivin, and cyclin D1. Morusin also decreased the expression of the stemness markers Oct4 and Nanog. The study showed the possible mechanisms by which morusin acts on CCSCs. It is known that almost all cases of colon cancer include activating mutations in the Wnt/-catenin pathway, and that this system is essential for CCSCs (Zhou et al., 2021). Thus, morusin may be viewed as a cutting-edge anticancer drug that targets colorectal carcinoma.

##### Nasopharyngeal carcinoma

Nasopharyngeal carcinoma (NPC) has an incidence rate of 50 per 100,000 persons and is prevalent in some regions, including Southern China and Southeast Asia (Wong et al., 2021). Radiotherapy is the main curative treatment for nasopharyngeal cancer. Although Intensity-Modulated Radio Therapy (IMRT) is the preferred method for treating locally advanced illness, chemotherapy combined with IMRT is an additional alternative. NPC often has a favorable prognosis than all other head and neck malignancies combined, and IMRT has had positive outcomes with more than 85% control of tumor spread. However, distant or lymph node metastases continue to be the most difficult circumstance (Lee et al., 2019). Therefore, improved systemic drugs that can prevent NPC metastasis should be sought. An earlier study found that poor survival and lymph node metastases were caused by greater plasma MMP-2 expression in NPC patients (Sakorafas et al., 2004). According to a recent study, morusin can prevent human NPC HONE-1, NPC-39, and NPC-BM cells from migrating and invading. These findings showed that morusin is effective at preventing NPC invasion and metastasis (Huang et al., 2020). In addition to being a tumor marker in NPC, MMP-2 is also linked to lymph node metastases and a poor prognosis (Liu et al., 2020). This indicates that MMP-2 is a significant contributor to metastasis in NPC. Results showed that morusin reduced NPC cells’ ability to migrate and invade by preventing the expression of the MMP-2 protein. Previous research has shown that early cancer metastasis prevention can be achieved by inhibiting MMP activity (Chaudhary et al., 2013). Morusin could therefore be used as a potential cancer treatment drug.

##### Breast cancer

Although there are several options for therapy available for individuals with primary breast cancer, including surgery, radiation, chemotherapy, targeted therapy, and hormone therapy, there are still many restrictions in breast cancer treatment due to the complicated disease variables (Trayes and Cokenakes, 2021). Therefore, improvements in research and our understanding of the underlying genetic causes of breast cancer may aid in lowering the disease’s incidence rate. Therapeutic resistance results from malignant tumors’ capacity to evade apoptosis and other death signals. The most popular method of avoiding apoptosis in malignancies such as breast cancer is the downregulation of apoptosis-associated proteins like Bcl-2-associated-x protein (Bax) and survivin (Singh et al., 2019). In a recent study, the potential of the drug morusin as a treatment for breast cancer was assessed in MCF-7, MDA-MB-231, MDA-MB-157, and MDA-MB-453 cell lines (Kang et al., 2017). To see if morusin may be used as a therapeutic component in the therapy of melanoma, cytotoxicity tests were performed on human breast cancer cells (Kang et al., 2017). Morusin was found to cause apoptosis in breast cancer cells, but at the same dose, the apoptotic rate was lower than the rate of necrosis. These findings suggest that morusin may cause necrosis and autophagy in breast cancer cells, in addition to other forms of cell death. By inhibiting the anti-apoptotic protein survivin, the study showed that morusin is a possibility for an anti-cancer medication to treat breast cancer (Kang et al., 2017). The fact that morusin increased Bax expression in breast cancer cells demonstrates its ability to target both pro- and anti-apoptotic signals, which may improve anticancer effectiveness. In light of these results, morusin deserves further study as a potential effective anticancer medication for the therapy of melanoma.

##### Gastric cancer

Despite a general downward trend in the frequency of the disease over the course of the last few decades, gastric cancer remains the third leading cause of death due to the disease. The development of chemo-preventive medications that can act as different tactical choices and the identification of effective crude drugs are essential for treating gastric cancer (Joshi and Badgwell, 2021). In a study, different concentrations of morusin were applied to gastric cancer cell lines MKN45 and SGC7901. According to the results of investigations using MTT as well as BrdU, morusin was able to inhibit the growth of gastric cancer cells in a dose-dependent way (Wang et al., 2017). The cell cycle progression of gastric cancer cells was halted by morusin due to its capacity to inhibit cyclin-dependent kinases and cyclins. Morusin decreased the expression of cell cycle-related proteins including Ki67 in xenograft tumor tests, which led to a decrease in the volume and size of gastric tumor cell aggregates *in vivo*. Morusin significantly attenuated the transcription of c-Myc in two cell lines, both at the mRNA and protein levels, and the effects were dosage and time dependent. Examining gastric tumors extracted from nude mice treated with morusin yielded the same result (Wang et al., 2017). Morusin, as shown here, inhibits c-Myc-dependent proliferation and tumor development in gastric cancer, suggesting its potential utility as neoadjuvant chemotherapy or as an alternative treatment for patients with this disease.

##### Prostate cancer

Prostate cancer ranks as one of the most often diagnosed malignancies in males. Despite treatment, many patients with intermediate or high-risk localized, locally progressed, or metastatic cancer present with the disease appear with an indolent course without any threat to mortality (Teo et al., 2019). The third most frequent reason for cancer-related death among males in the US is prostate cancer (Ahmad et al., 2021). Most prostate cancers are testosterone-dependent in their early stages, and these tumours can be treated with surgery, radiation, and androgen suppression. However, for advanced and metastatic prostate cancer, these treatments are ineffective (Teo et al., 2019). As a result, phytochemicals have been found to be useful in treating prostate cancer that has progressed to an advanced stage. Morusin reduced cell survival in prostate cancer cell lines DU145, PC3, M2182, and LNCaP, but had no effect on healthy prostate cells in a cytotoxicity experiment meant to evaluate the drug’s potential as a therapy for prostate cancer (Lim et al., 2015). The SRC/JAK2/STAT3 signaling pathway was demonstrably blocked by morusin by preventing their phosphorylation, which led to STAT3’s loss of nuclear accumulation and transcriptional activity. This STAT3 suppression decreased the expression of genes encoding for cell cycle regulator proteins including c-Myc and Cyclin D1 and anti-apoptotic proteins like Bcl-xL, Bcl-2, and survivin (Lim et al., 2015). These findings imply that morusin may be a promising anti-cancer treatment for prostate cancer because it causes apoptosis via suppressing STAT3 by inducing SHP1 in prostate cancer cells.

##### Glioblastoma multiforme

The most dangerous and aggressive primary brain tumor in humans, glioblastoma multiforme (GBM), has a terrible prognosis. An increasing number of studies has revealed that cancer stem cells (CSCs) are a subset of GBM cells that are resistant to standard chemo- and radiation and are therefore thought to be responsible for the disease’s progression and recurrence despite treatment (Wu et al., 2021b). The development of tumorus, their recurrence, and their resistance to standard therapy are all caused by GBM cancer stem cells (GSCs). GBM treatment is regarded as a significant therapeutic challenge. Consequently, it may be very beneficial to develop novel cancer therapeutics by studying and producing drugs that block GSC activity and induce them to transdifferentiate or specialize into adult cell lines (Janjua et al., 2021). Morusin inhibited the formation of GSC neurospheres and the growth of GSCs in research involving GSCs and GBM cells. Protein levels of PPARγ, Bax, and caspase-3 were markedly increased in GSCs exposed to morusin, whereas Bcl-2 expression was markedly decreased. Morusin was thought to possibly have a role in controlling the cell cycle and apoptosis. Following morusin treatment, stemness proteins such as CD133, nestin, Oct4, and Sox2 were significantly downregulated in both GSCs and tumor tissues. Morusin has a potent growth-inhibiting effect on human GSCs both *in vitro* and *in vivo*, and its mode of action may include the attenuation of GSC stemness, the differentiation of GSCs into adipocytes, and the induction of death (Guo et al., 2016). Since it may be the first therapeutic medication to specifically target GSCs for the treatment and/or prevention of human GBM, it warrants further investigation.

#### Mechanism of antitumor action of morusin

##### Inhibition of proliferation

Myc genes are essential nucleus transcription factors and belong to the family of basic helix-loop-helix (b-HLH) DNA-binding proteins (Elbadawy et al., 2019). To a large extent, Myc regulates the processes of tumorigenesis, cell growth, maturation, the biogenesis, metabolic, viability, and death (Hua et al., 2019; Wang et al., 2019). c-Myc expression is normally regulated by interactions with the extracellular matrix and growth factors. Dormant cells express c-Myc, but only at low levels. When a cell begins the cell cycle, c-Myc expression swiftly rises. Then, during cell division, its expression gradually decreases until it returns to its initial, low level (Reyes-González and Vivas-Mejía, 2021). Morusin inhibited the expansion of gastric cancer cells by decreasing the activity of cyclin-dependent kinases (CDKs) CDK2, CDK4, and cyclins Cyclin D1, and Cyclin E1. In two different gastric cancer cell lines MKN45 and SGC7901, morusin treatment significantly reduced c-Myc expression at the mRNA and protein levels in a time- and dose-dependent manner (Wang et al., 2017). Although c-Myc transcription is tightly controlled during healthy cellular growth, it is frequently uncontrolled in human malignancies. Overexpression of c-Myc in approximately 40% of stomach tumours is a common factor in poor prognosis (Panda et al., 2020). c-Myc has been shown to control a vast array of genes involved in processes as diverse as metabolism, ribosome biogenesis, protein synthesis, and mitochondrial function. Two cell cycle-related genes, CDKs and Cyclins, were the first “c-Myc target genes” to be discovered, and their expression levels change in response to c-Myc activation (Chaudhuri et al., 2020). Overexpression of c-Myc led to a rise in the protein levels of cell cycle regulators CDK2, CDK4, Cyclin D1, and Cyclin E1. According to results from a cell cycle analysis, overexpression of c-Myc was able to counteract the inhibition of cell proliferation caused by morusin. Cells treated with morusin showed increased protein production of cyclin-dependent kinases and CDKs when c-Myc was overexpressed. Research indicates that through decreasing c-Myc levels, morusin inhibits the development and spread of tumours in stomach cancer (Wang et al., 2017).

##### Apoptosis induction

Apoptosis is a kind of controlled cell death that may occur under both typical and pathological conditions (Neophytou et al., 2021). The ultimate goal of many chemotherapeutic agents is to induce apoptosis in cancer cells (Pang et al., 2021). Morusin can trigger apoptosis in cell lines MKN45 and SGC7901, according to a recent study (Wang et al., 2020). Apoptosis signalling systems may be classified as either intrinsic or extrinsic. The intrinsic route is sometimes referred as the mitochondrial pathway owing of its association with mitochondria (Jan and Chaudhry, 2019). It was discovered that morusin induced apoptosis by means of the internal mitochondrial lethal mechanism (Wang et al., 2020). Caspase-9 breakage, MMP loss, cytochrome c release into the cytoplasm, Bax upregulation, and Bcl-2 downregulation were all indicative of this. When cells undergo shocks to the outer membrane, autophagy often occurs before apoptosis, another kind of regulated cell death. Two common autophagy markers are LC3-II and SQSTM1 (Smith and Macleod, 2019). After 30 min of morusin treatment, the protein levels of LC3-II and SQSTM1 were demonstrated to be inversely regulated, with the former rising while the latter falling as a result of autophagy (Wang et al., 2020). ERK and JNK, two members of the mitogen-activated protein kinase (MAPK) family, play crucial roles in regulating cell cycle progression, propagation, and longevity (Guo et al., 2020). Regulating cell proliferation and death is mostly dependent on the PI3K-Akt mechanism. Morusin dephosphorylated Akt while phosphorylating JNK, ERK, and JNK. NF-κB was demonstrated to be inhibited as an Akt downstream target in a suitable manner. Using the relevant inhibitors for each pathway, it was discovered that ERK, JNK, and morusin-induced apoptosis and autophagy include Akt pathways. According to multiple studies, oxidative stress is just one of the many stress stimuli that all of the pathways outlined above are responsive to (Rezatabar et al., 2019). After receiving morusin treatment, ROS production was shown to have greatly risen. Morusin-induced apoptosis and autophagy were inhibited by co-treatment with NAC (*N*-acetyl-L-cysteine), indicating that ROS was a contributing component in their induction. Morusin successfully reduced cell viability by triggering endogenous apoptosis and boosting autophagy flux. Apoptosis and autophagy were induced by MAPK ERK, JNK activation and PI3K/Akt inhibition. One potential mechanism of action for morusin is the augmentation of intracellular ROS production that is implicated in the control of PI3K/Akt and MAPK.

##### Inhibition of cancer cell invasion and metastasis

Cancer is characterized by a number of features, including resistance to cell death, immune system avoidance, malignant cells aggravation, and activated metastasis and invasion. Initiating invasion and metastasis are crucial processes because regional and distant metastasis are the most significant and challenging circumstances for cancer patients (Esposito et al., 2021). The breakdown of the vascular endothelium’s basement membrane and extracellular matrix is one of the many molecular interactions that make up metastatic process. The basement membrane and ECM’s constituent parts are both degraded in a significant way by the MMP family (Abdel-Hamid and Abass, 2021). MMP-2 and MMP-9 are the most significant proteins in metastasis, out of the over 25 MMP members that have been found. At the angiogenic switch, the first step of tumor vascularization, MMP-2 and MMP-9 have been shown to be crucial (Li et al., 2022). According to recent findings, morusin suppressed the expression of MMP-2 but not MMP-9 in a concentration-dependent way in NPC HONE-1, NPC-39, and NPC-BM cell lines. Loss of angiogenic capacity and inhibition of tumor cell motility are the outcomes of MMP-2 downregulation. MMP-2 is well known for degrading ECM as well as being a tumor marker for head and neck cancer with a bad prognosis. According to studies, morusin blocks the ability of cancer cells to migrate and invade by preventing the expression of the MMP-2 protein (Huang et al., 2020). Previous research has shown that early MMP activity inhibition can be used to prevent cancer metastasis, and morusin may be a promising cancer therapeutic agent. Mitogen-activated protein kinases (MAPKs) are a family of serine-threonine protein kinases that play an essential role in cell differentiation, migration, and death. Proteolytic phosphorylation of ERK1/2 was inhibited by morusin (Huang et al., 2020). According to a prior study, patients with carcinoma had high levels of p-ERK1/2 and Ki-67 expression, and the ERK1/2 signaling pathway plays a role in both cancer growth and apoptosis (Chen et al., 2017). In a different study, pinostilbene hydrate suppressed the epithelial-mesenchymal transition and downregulated MMP-2 to prevent tumor cell migration and invasion (Tseng et al., 2019). A prospective candidate for chemoprevention or adjuvant therapy, morusin administration greatly reduced the phosphorylation of ERK1/2, which regulates the metastatic processes of many malignancies.

##### Anti-angiogenic properties

**FIGURE 4:**
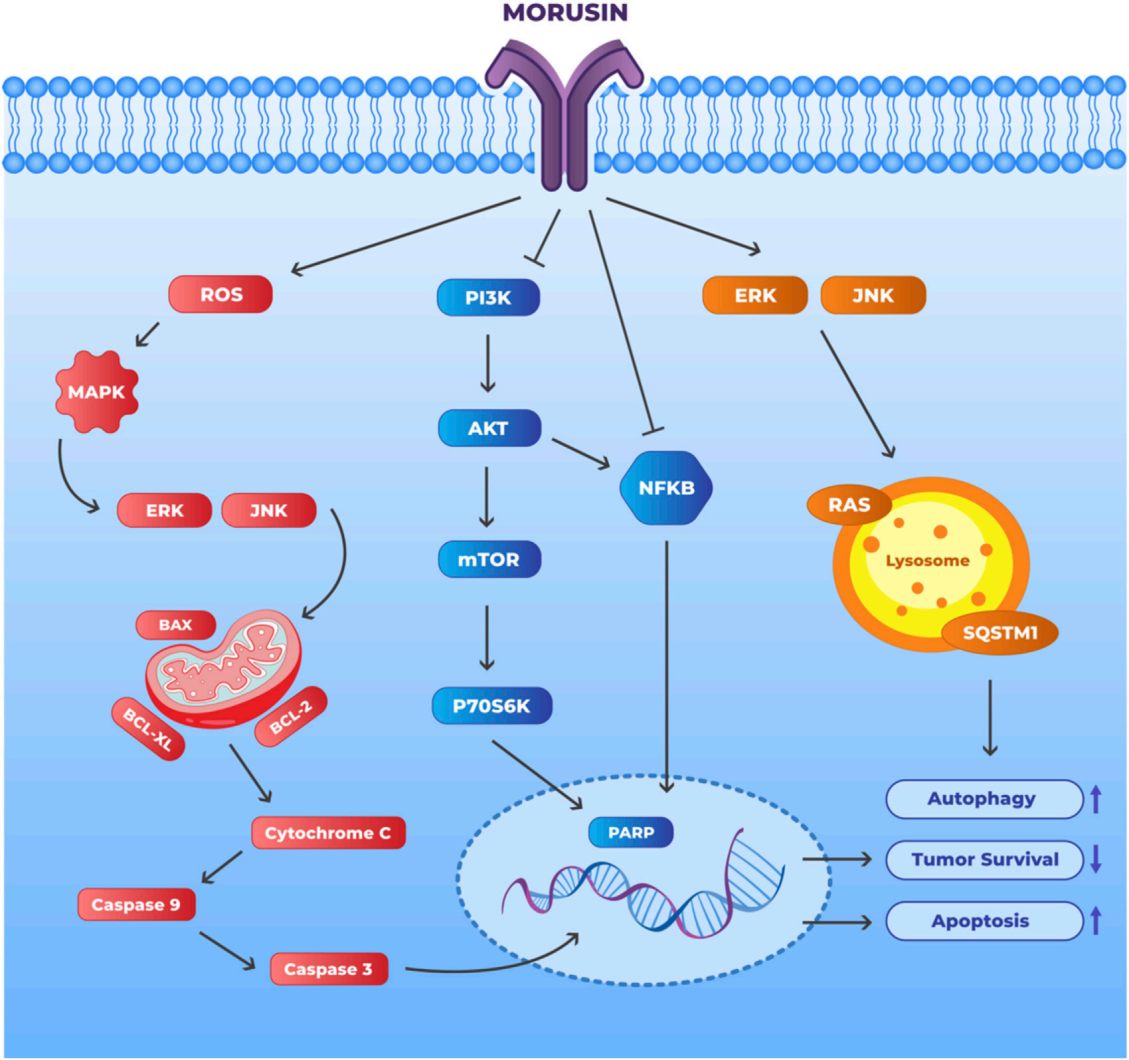
Mechanism of action of morusin to promote apoptosis and autophagy and inhibit tumor survival. Morusin may have potential as a cancer therapeutic agent through its ability to target multiple signaling pathways. The black arrows depict the direct effect of morusin treatment on the protein and the process. Morusin upregulates apoptosis and autophagy in tumor cells through the activation of the ERK/JNK pathway as well as increasing ROS within the cells. Morusin also downregulates tumor survival through inhibiting NF-Κb and PI3K. (Abbreviations: BCL2: B-cell lymphoma-2, ERK: Extracellular signal-regulated kinase, JNK: c-Jun N-terminal Kinase, MAPK: Mitogen-activated protein kinases, PI3K: Phosphoinositide 3-kinases, NF-Κb: Nuclear factor kappa B, PARP: Poly (ADP-ribose) polymerase and mTOR: mammalian target of rapamycin.).

Malignancies, particularly solid tumorus, rely heavily on aberrant angiogenesis for their development and spread. VEGF is a crucial regulator of vascular permeability for the control of angiogenesis and encourages EC migration and proliferation (Lugano et al., 2020). Several Chinese herbal ingredients have proven to have anti-angiogenesis properties that specifically target VEGF-induced angiogenesis. Pedicularioside G, a phenylpropanoid glycoside, and resveratrol, which could lower VEGF synthesis in HepG2 cells, have both been shown to inhibit tumor cell propagation and motility (Mu et al., 2008; Zhang and Yang, 2014; Frezza et al., 2019). According to reports, morusin suppresses VEGF expression in Huh7 and Hep3B cells. Receptor tyrosine kinase, particularly VEGFR2, a significant VEGF signal transducer, specifically responds with VEGF. As VEGFR2 transcription was downregulated in tumor cells after morusin treatment, it seemed possible that the medication addressed the vascular endothelial growth factor (VEGF)/VEGFR2 signaling pathway, suggesting that morusin acted as an angiogenesis inhibitor (Gao et al., 2017). The antiangiogenic activity of morusin has been shown *in vivo*. CD34, a marker for microvessels, is considered to be sensitive and specific for detecting hepatocellular carcinoma. Expression of CD34 in tumor tissues was downregulated after morusin treatment, which correlated with less microvascular density (MVD) in the tumor tissues that had been treated with morusin (Gao et al., 2017). MVD is the compression of small blood vessels within the tumor due to abnormal angiogenesis taking place within the tumor, leading to poor oxygen and nutrient supply. MVD forces the tumor to quickly adapt to low oxygen conditions and become more invasive and is associated with poor prognosis as well as resistance to therapy (Chen et al., 2013). Morusin was shown to lower MVD in tumors. These data point to the possibility that morusin has antiangiogenic properties in cell culture and in animal models. Several primary tumorus and cancer cell lines are known to contain constitutively activated STAT3 (Zou et al., 2020). IL-6 has been linked to constitutive or aberrant STAT3 activation in many types of cancer for quite some time (Lin et al., 2020). Researchers have discovered that activated STAT3 promotes angiogenic component target signaling pathways and regulates angiogenic factor expression. Inhibition of IL-6 and phosphorylated STAT3 synthesis may be responsible for morusin’s effectiveness in killing human HCC cells and halting angiogenesis (Gao et al., 2017). Therefore, the IL-6/STAT3 signaling pathway is now considered a potential therapeutic target for a wide variety of diseases. Morusin inhibits the development of human cancers in test tubes and living animals by increasing apoptosis and decreasing angiogenesis. The mechanism may function to dampen the IL-6/STAT3 signaling pathway. Therefore, further research into morusin is warranted because of the potential anti-angiogenic effects of morusin in the treatment and/or management of malignancies. Figure 4 illustrates the morusin mechanism in cancer.

##### Potential *in vivo* and *in vitro* studies

We did not have direct data from controlled clinical trials of the anti-cancer properties of flavonoids until recently. However, at this time, clinical studies are beginning to bridge the gap between actual clinical practice and tests conducted in a research laboratory. Researchers are attempting to investigate the potential of flavonoids to prevent and treat cancer in clinical settings, where some flavonoids have qualified for various stages of clinical studies. Some flavonoids, including green tea’s catechin and epigallocatechin-3-gallate (EGCG), have shown promising results, and are now in phase 2 cancer preventative and therapeutic research studies (Sharma et al., 2022). Rich in flavonoids, watercress is undergoing a phase 3 clinical research and showing remarkable results for chemoprevention (Sharma et al., 2022). However, it is quite unfortunate that none of the flavonoids have, to date, been able to effectively treat or prevent cancer, which may be related to flavonoids’ physiological limits. Flavonoids are a significant research topic worldwide due to their accessibility in our daily food, their anticancer qualities, and the fact that they can be produced at comparatively inexpensive costs. In order to boost the bioavailability of flavonoids, researchers are constantly looking for new therapeutic strategies. These strategies could involve improving the penetration of flavonoids across cytoplasmic membrane or increasing the degree to which they are soluble in water. The use of hydrophilic, inert transport molecules that are converted into nontoxic intermediates is another method of functionalizing healthful compounds that are poorly soluble under physiological circumstances. (Panek-Krzyśko and Stompor-Gorący, 2021).

One of the flavonoids generated from the *Morus* plant, morusin, has received attention for having a superior antioxidant potential compared to other flavonoids. Despite the fact that the processes and clinical evidence *in vivo* have not yet been extensively analyzed, it has undergone extensive testing in *in-vitro* systems for its multifarious potential against human illnesses such as cancer, immunological dysfunction, and metabolic disorders. Regrettably, the usage of flavonoids in the cancer therapy is not effective due to their weak solubility, limited absorption, and quick metabolism (Kumar and Pandey, 2013; Cassidy and Minihane, 2017). Modern nanotechnology may be applied in this context. By using nanocarriers, flavonoids’ bioavailability can be increased. Flavonoid nanoparticles may exhibit anticancer action against lung cancer cells, melanoma cells, MCF-7 breast cancer cells, HepG2 liver cancer cells, or colorectal cancer cells, according to *in-vitro* and *in-vivo* studies (Wu et al., 2016; Dobrzynska et al., 2020). Flavonoid nanocarriers come in a variety of forms and are now used in cancer treatment. These could be solid lipid nanocarriers, metallic nanoparticles, nanocapsules, or polymeric nanoparticles (Katopodi and Detsi, 2021; Khan et al., 2021). The FDA has cleared the use of PLGA nanoparticles, a polymer that was developed by researchers and is safe for people to consume, biocompatible, recyclable, and easy to modify. For the treatment of glioblastoma, they used a nano formulation composed of PLGA nanoparticles loaded with morusin and conjugated to chlorotoxin (Agarwal et al., 2019). It has been discovered that average healthy neural cells are less successful than cancer cells at entrapping the medication molecule. In addition, therapy with altered PLGA-MOR-CTX nanoparticles led to increased caspase activation, cytoskeletal disruption, inhibition of MMP-2 action, and ROS production in the tumor tissue (Agarwal et al., 2019). The same nanoparticles loaded with morusin can prove to be beneficial in other cancers and *in-vivo* as well as *in-vitro* studies are required to assess the potential use of PLGA-MOR-CTX formulation for various cancers. Adjuvant therapies with morusin provide a promising avenue of cancer therapeutic research.

Numerous cellular processes in a cancer cell are dynamically remodelled throughout time, providing diverse benefits including treatment resistance. In response to morusin treatment, cancer cells promote autophagy and stress granules (SG) formation, desensitising the apoptotic signals produced by morusin treatment (Choi et al., 2020). The “shield routes” by which tumours resist various drug regimens have been proposed to be autophagy and SGs. This notion suggested the co-treatment of morusin and the inhibitors as a therapeutically relevant strategy for treating cancers that are resistant to treatment, along with numerous biochemical studies demonstrating that morusin and repressors of autophagy and the formation of SG enhanced each other’s antitumor activity. It is crucial to take into account if these resistance mechanisms still hold true for various cancer subtypes *in vivo*. In order to advance toward clinical trials, the potential of morusin as an alternative therapeutic strategy for cancers that are chemo-resistant needs to be investigated in animal models.

## Conclusion

Studies on the possible therapeutic effects of the flavonoid component morusin have focused on its antioxidant, anti-inflammatory, and anticancer characteristics. Researchers have looked at the anti-inflammatory properties of morusin. Morusin may have anti-inflammatory actions and can block the generation of pro-inflammatory cytokines, according to certain studies. Morusin has been demonstrated to have antioxidant capabilities in addition to its potential anti-inflammatory and anti-cancer benefits. Several researches have examined morusin’s potential to inhibit the growth of cancer cells. Particularly in breast and colon cancer cells, morusin may trigger cell apoptosis (programmed cell death). Additionally, it has been demonstrated to impede the invasion and migration of cancer cells, which may aid in halting the spread of the disease to other body regions. Even though morusin has a great therapeutic potential, additional research is required to completely comprehend its effects and figure out the best method to apply it as a treatment.

## Figures and Tables

**Table:** 

**5-LOX**	Arachidonate 5-lipoxygenase
**AGE**	Advanced glycation end products
**AKT**	Ak strain transforming
**AMPK**	AMP-activated protein kinase
**Ap2**	Adipocyte protein 2
**ATBS Assay**	2,2′-azino-bis(3-ethylbenzothiazoline-6-sulfonic acid assay
**BAX**	Bcl-2-associated X protein
**BCL-2**	B-cell lymphoma 2
**CDK**	Cyclin-dependent kinases
**CMS**	Chinese Medicine System
**C-myc**	Cellular myelocytomatosis
**CTX**	Chlorotoxin
**COX**	Cyclooxygenase
**CRC**	Colorectal Cancer
**CSC**	Cancer stem cells
**CYPA34**	Cytochrome P450 3A4
**EGCG**	Epigallocatechin gallate
**ERK**	Extracellular signal-regulated kinase
**GABA**	Gamma-aminobutyric acid
**GBM**	Glioblastoma Multiforme
**GPDH**	Glycerol-3-phosphate dehydrogenase
**GSK**	Glycogen synthase kinase
**HCC**	Hepatocellular carcinoma
**HIF-1**	Hypoxia-Inducible Factor 1
**HIV**	human immunodeficiency virus
**IL6**	Interleukin 6
**IUPAC**	International Union of Pure and Applied Chemistry
**LC3**	Microtubule-associated protein 1A/1B-light chain 3
**LOX**	lipoxygenase
**LPS**	lipopolysaccharide
**JNK**	Jun N-terminal kinase
**MAPK**	Mitogen-activated protein kinase
**MMP**	Matrix metalloproteinase
**MTOR**	mammalian target of rapamycin
**MTT**	(3-(4, 5-dimethylthiazolyl-2)-2, 5-diphenyltetrazolium bromide)
**MVD**	Microvascular decompression
**NF-κB**	Nuclear factor kappa B
**NPC**	Nasopharyngeal carcinoma
**OCT4**	Octamer-binding transcription factor 4
**PGLA**	Poly-lactic-co-glycolic acid
**PI3K**	phosphoinositide 3-kinase
**PPAR**	Peroxisome proliferator-activated receptors
**RCC**	Renal cell carcinoma
**SG**	Stress Granules
**SHP-1**	Src homology 2 domain-containing protein tyrosine phosphatase 1
**SQSTM1**	Sequestosome 1
**STAT3**	Signal transducers and activators of transcription 3
**UDP-glucuronosyltransferase**	Uridine 5′-diphospho UDP-glucuronosyltransferase
**VEGF**	Vascular endothelial growth factor
**VEGFR**	Vascular Endothelial Growth Factor Receptor
**Wnt**	Wingless-related integration site
